# Physical exercise in patients with testicular cancer treated with bleomycin, etoposide and cisplatin chemotherapy: pulmonary and vascular endothelial function—an exploratory analysis

**DOI:** 10.1007/s00432-023-05469-5

**Published:** 2023-10-27

**Authors:** Gabriela G. F. van der Schoot, Harm L. Ormel, Nico-Derk L. Westerink, Johan B. Wempe, Joop D. Lefrandt, Anne M. May, Aline H. Vrieling, Coby Meijer, Jourik A. Gietema, Annemiek M. E. Walenkamp

**Affiliations:** 1 Department of Medical Oncology, University Medical Center Groningen, University of Groningen, Hanzeplein 1, 9713 GZ Groningen, The Netherlands; 2 Department of Pulmonary Medicine, University Medical Center Groningen, University of Groningen, Groningen, The Netherlands; 3 Department of Vascular Medicine, University Medical Center Groningen, University of Groningen, Groningen, The Netherlands; 4 Julius Center for Health Sciences and Primary Care, University Medical Center Utrecht, Utrecht University, Utrecht, The Netherlands; 5 Department of Rehabilitation Medicine, University Medical Center Groningen, University of Groningen, Groningen, The Netherlands

**Keywords:** Cancer, Bleomycin, Cisplatin, Pulmonary toxicity, Vascular endothelial dysfunction, Physical exercise

## Abstract

**Purpose:**

Bleomycin, etoposide, and cisplatin combination chemotherapy (BEP) improves the survival of patients with testicular cancer, but is associated with potentially life-threatening toxicities like pneumonitis and thromboembolic events. This study explored the effects of physical exercise in patients with testicular cancer during or after BEP-chemotherapy on pulmonary and vascular endothelial toxicity.

**Methods:**

In this post hoc analysis of a multicenter randomized clinical trial (NCT01642680), patients with metastatic testicular cancer scheduled to receive BEP-chemotherapy were randomized to a 24-week exercise intervention, initiated during (group A) or after BEP-chemotherapy (group B). Endpoints were pulmonary function (forced vital capacity (FVC), forced expiratory volume in one second (FEV1), lung transfer-coefficient and transfer factor for carbon monoxide (KCO, DLCO) and markers of vascular endothelial dysfunction (von Willebrand factor (vWF) and factor VIII).

**Results:**

Thirty patients were included. Post-chemotherapy, patients declined less in FVC, FEV1 and DLCO in group A compared to group B. Post-chemotherapy, vWF and factor VIII were significantly lower in group A compared to group B. After completion of exercise, started either during BEP-chemotherapy or thereafter, no between-group differences were found. At 1-year post-intervention, significant between-group differences were found in favour of group A in DLCO and KCO.

**Conclusions:**

Patients who exercised during BEP-chemotherapy better preserved FVC, FEV1 and DLCO, measured directly post-chemotherapy and 1-year post-intervention (DLCO, KCO). This coincided with less increase in vWF and factor VIII measured directly post-chemotherapy. These data support a beneficial role of a physical exercise intervention during BEP-chemotherapy on pulmonary and vascular damage in patients with testicular cancer.

**Trial registry:**

Optimal Timing of Physical Activity in Cancer Treatment (ACT) Registry URL: https://clinicaltrials.gov/ct2/show/NCT01642680. Trial registration number: NCT01642680.

**Supplementary Information:**

The online version contains supplementary material available at 10.1007/s00432-023-05469-5.

## Introduction

The introduction of bleomycin, etoposide and cisplatin combination chemotherapy (BEP) has improved the long-term survival of patients with metastatic testicular cancer to more than 90% (Hanna and Einhorn 2014). Unfortunately, BEP chemotherapy is associated with toxicities like bleomycin-induced pneumonitis (BIP), fatal in up to 3% of patients, and thromboembolic events (Watson et al. 2018; Sleijfer 2001; Haugnes et al. 2015).

Endothelial damage of the small vessels in the lung plays an important role in the development of bleomycin-induced pulmonary toxicity (Sleijfer 2001). Bleomycin binds to DNA, directly leading to DNA damage and indirectly generating reactive oxygen species (Sleijfer 2001; Della Latta et al. 2015). The bleomycin hydrolase enzyme degrades bleomycin, but the lungs might be more prone to develop toxicity due to a low concentration of bleomycin hydrolase in pulmonary tissue (Crnovcic et al. 2018). Bleomycin activates vascular endothelial cells to secrete inflammatory cytokines and profibrotic mediators like transforming growth factor–β (Liu 2008; Leach et al. 2013). These mediators stimulate the recruitment and activation of fibroblasts and can lead to lung fibrosis development (Sleijfer 2001). In endothelial cells of lung tissue with bleomycin-induced fibrosis, levels of von Willebrand factor (vWF) and plasminogen activator inhibitor type 1 (PAI-1) are elevated, both markers of vascular endothelial dysfunction (Marullo et al. 2013; Leach et al. 2013; Della Latta et al. 2015; Kato et al. 2018). Vascular endothelial dysfunction is associated with the development of thromboembolic events in patients with testicular cancer, both during and after treatment with bleomycin and cisplatin combination chemotherapy (Nuver et al. 2005; Haugnes et al. 2012; Lubberts et al. 2016; Haugnes et al. 2015).

Evidence mounts that engaging regularly in moderate-to-vigorous intensity aerobic physical exercise is beneficial for vascular endothelial function by increasing nitric oxide availability (Thompson et al. 2003; Fiuza-Luces et al. 2018; Ignarro 2002; Forstermann and Münzel 2006; Ozemek et al. 2018). Also, physical exercise enhances the production of antioxidant enzymes like glutathione peroxidase and mitochondrial superoxide dismutase, which counteract oxidative stress (Domenech and Viña 2008; Sties et al. 2018).

Based on these data, we hypothesized that structured physical exercise during BEP-chemotherapy attenuates pulmonary toxicity and vascular endothelial dysfunction in patients with metastatic testicular cancer. This exploratory analysis aimed to investigate the effect of a physical exercise intervention performed during or after BEP-chemotherapy on pulmonary function and markers of vascular endothelial dysfunction, measured before and post-chemotherapy, post-intervention, and 1-year post-intervention.

## Materials and methods

### Study design and participants

**Fig. 1:**
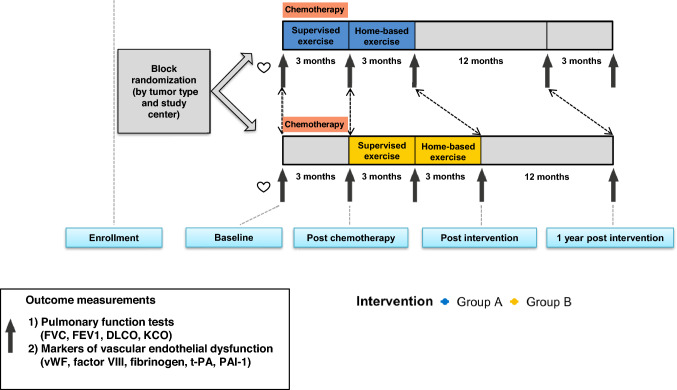
Design of the tailored physical exercise intervention during or after BEP-chemotherapy**.** *Group A* initiated the intervention during chemotherapy, *Group B* initiated the intervention after chemotherapy, *DLCO* carbon monoxide diffusing capacity, *KCO* carbon monoxide transfer coefficient, *FVC* forced vital capacity, *FEV1* forced expiratory volume-one second, *vWF* von Willebrand factor, *PAI-1* plasminogen activator inhibitor type 1 antigen, *t-Pa* tissue-type plasminogen activator antigen

This is an exploratory post hoc analysis of the multicenter, two-armed, randomized clinical ACT-trial. A detailed method description of the ACT-trial has been reported elsewhere (van der Schoot et al. 2022). In brief, the protocol was amended in March 2016 to perform pulmonary function tests in patients with testicular cancer treated with BEP-chemotherapy. For the present exploratory analysis, patients with metastatic testicular cancer treated with BEP-chemotherapy were eligible. Inclusion criteria were normal blood count and regulated blood pressure, resting respiratory rate < 20 breaths/min, heart rate 50–100 beats/min and a left ventricular ejection fraction ≥ 50%. Exclusion criteria were infections requiring antibiotics, critical organ impairment or uncontrolled symptoms due to malignancy, no recovery from earlier surgical intervention, inability to travel to the rehabilitation center, recent cardiovascular event (< 6 months) and a cognitive disorder or emotional instability (e.g. an active psychosis or severe anxiety disorder, which might affect participation in the training program). Patients were randomized using a concealed computerized randomization program in a 1:1 ratio to an exercise intervention initiated ‘during’ (group A) or ‘after’ (group B) chemotherapy (Fig. 1). All patients gave written informed consent.

### Procedures

The intervention consisted of two components: 12 weeks of supervised exercise followed by 12 weeks home-based unsupervised exercise. The supervised exercise consisted of aerobic exercise training thrice a week, resistance exercise training twice a week and optionally once per week leisure sports game activities. The intensity of aerobic exercise training was prescribed based on training heart rate (HR), calculated with the Karvonen formula (training HR = (HR_max_ – HR_rest_) × intensity (%) + HR_rest_), using the HR_max_ and HR_rest_ from cardiopulmonary exercise testing assessed at baseline (Karvonen and Vuorimaa 1988). The intensity increased from 50% (week 1–4), 60% (week 5–6), 70% (week 7–10) and 75% (week 11–12). Initial resistance was fixed on 50% of the 1-repetition maximum during the first week. In the following weeks, resistance increased by 5–10%. During the home-based program, patients were asked to continue aerobic exercise training by walking, running or cycling and to fill out a training log for adherence to the prescribed exercise. Additional information about the training schedule, including an exemplar exercise schedule can be found in the Supplementary Information and Supplementary Table 1).

### Outcomes

Patients visited the outpatient clinic before the start of chemotherapy, directly post-chemotherapy, post-intervention and 1-year post-intervention (Fig. 1). Patient characteristics were obtained from medical records: tumour characteristics, chemotherapy dosages, medical history, medication and cardiovascular risk factors. Also, data on BIP risk factors (age at onset of chemotherapy, cumulative dose of bleomycin, pulmonary metastases, smoking status and renal function (glomerular filtration rate using the Cockroft Gault formula) were collected from medical records. Adherence to the exercise intervention was defined as the number of attended physical exercise sessions divided by the number of prescribed exercise sessions. Serious adverse events (SAEs) were monitored until the final measurement at 1-year post-intervention.

Data on bleomycin pulmonary toxicity were recorded using the following classification (Nuver et al. 2005): (a) death due to BIP, (b) clinical or radiographic signs of BIP resulting in hospitalization, premature cessation of bleomycin administration, use of steroids, (c) clinical or radiographic signs of BIP after completion of treatment, use of steroids, (d) no signs of BIP. Pulmonary function was assessed by use of dynamic spirometry (Masterscreen PFT-PRO, CareFusion). It included the parameters forced expiratory volume in 1 s (FEV1), forced vital capacity (FVC) and the FEV1/FVC ratio. The lung transfer-coefficient for carbon monoxide (KCO) and transfer factor for carbon monoxide (DLCO) were corrected for hemoglobin. Pulmonary function parameters were expressed as raw values (in figures) and as a percentage of predicted normal values (in tables). Fasting blood samples were analysed for vWF, factor VIII, fibrinogen, tissue plasminogen activator (t-PA) and PAI-1.

### Sample size and statistical analysis

Descriptive statistics were used to analyse patient characteristics.

Intention-to-treat linear mixed model analyses were performed for within-group and between-group differences at three timepoints—directly post-chemotherapy, post-intervention and 1-year post-intervention and were adjusted for baseline values. We calculated the standardized effect size (ES) by dividing the adjusted between-group mean difference of the measurement directly post-chemotherapy, post-intervention and 1-year post-intervention using the pooled T0 standard deviation. An ES < 0.2 was considered ‘no difference’, ES between 0.2 and 0.5 small difference, ES between 0.5 and 0.8 moderate difference and ES > 0.8 substantial difference (Cohen 1988). A 95% confidence interval (CI) not including 0 indicates significance at a *P* level of < 0.05.

## Results

**Fig. 2:**
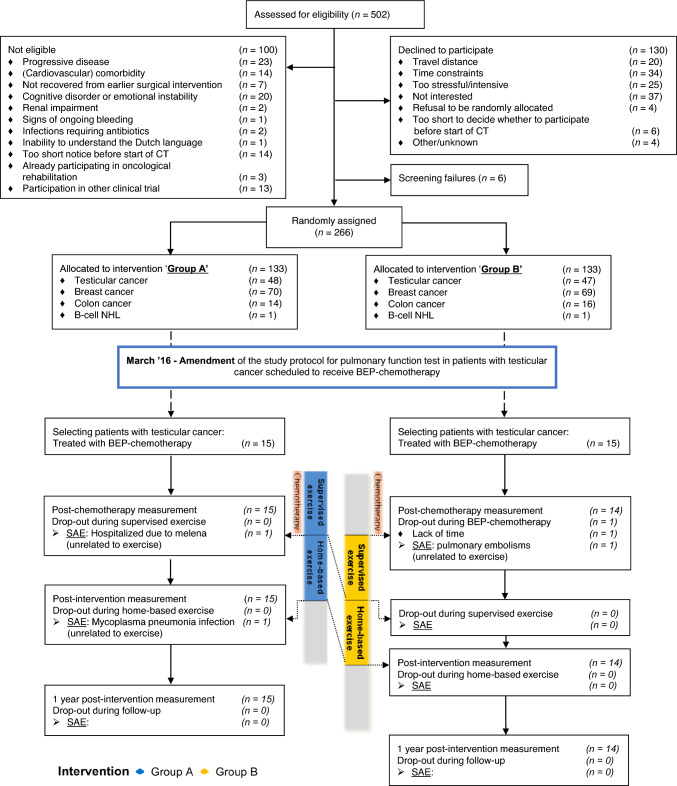
CONSORT diagram**.** *Group A* initiated the intervention during chemotherapy, *Group B* initiated the intervention after chemotherapy, *CT* chemotherapy, *n* number, *SAE* serious adverse event

From March 2016 to November 2018, 103 patients with testicular cancer were screened for eligibility for the ACT trial. There were no screen failures, 44 patients were ineligible, mostly due to other chemotherapy regimens than BEP or logistic reasons, 29 declined to participate and 30 patients were included. Of these patients, 15 were randomized in group A and 15 in group B (see CONSORT diagram, Fig. 2). The final data from the participating patients were collected in October 2020. Baseline and treatment characteristics were well balanced between groups and are listed in Table 1. The median age was 29.0 years (range 22–48) in group A, 30.0 years (range 22–43) in group B.

**Table 1:** Patient characteristics

	Group A (*n* = 15)	Group B (*n* = 15)
Age in years, median (range)	29.0 (22–48)	30.0 (22–43)
Weight in kg, median (range)	86.0 (71–116)	87.0 (59–113)
Royal Marsden staging		
II	14 (93%)	11 (74%)
III	–	2 (13%)
IV	1 (7%)	2 (13%)
IGCCCG prognosis group		
Good	15 (100%)	12 (80%)
Intermediate		3 (20%)
Lung metastases		
Yes	1 (7%)	2 (13%)
No	14 (93%)	13 (87%)
Cumulative bleomycin dose, USP Median (range)	270 (30–270)	270 (60–360)
Cumulative cisplatinum dose, mg/kg^2^ Median (range)	300 (300–595)	300 (300–400)
N courses of BEP		
3	13 (87%)	11 (73%)
4	2 (13%)	4 (27%)
GFR mL/min^2^ Median (range)	145 (109–221)	131 (98–185)
Cardiovascular risk factors at start of chemotherapy		
Smoking		
Never smoker	7/15	8/15
Ever smoker	8/15	7/15
Current smoker	5/15	4/15
Overweight^a^	5/15	6/15
BMI, median (range)	26.0 (20.5–21.5)	25.7 (19.3–30.3)
Hypertension^b^	2/15	3/15
Dyslipidemia^c^	12/15	11/14 (1 unknown)
Diabetes mellitus^d^	0 /15	0/14 (1 unknown)

*Group A* initiated the intervention during chemotherapy, *Group B* initiated the intervention after chemotherapy, *n* number, *kg* kilogram, *IGCCCG* international germ cell collaborative group risk classification, *USP* United States Pharmacopeia, *mg* milligram, *BEP* bleomycin, etoposide, cisplatin combination chemotherapy, *ml* milliliter, *min* minute, *m* meter

^a^Body mass index (BMI) > 25 kg/m^2^

^b^Blood pressure > 140/90 mm Hg (or using antihypertensive drugs)

^c^Dyslipidaemia (total cholesterol > 5.1 mmol/L, LDL > 2.5 mmol/L or using lipid lowering drugs)

^d^Fasting glucose ≥ 7 mmol/L or HbA1c > 6.5% (or using blood glucose lowering drugs)

The median adherence rate to the supervised physical exercise of patients from group A (*n* = 12) was 77.8% (range 47.2–100.0%), median adherence of patients from group B (*n* = 13) was 80.6% (range 5.6–125.0%), *P* = 0.743. From five patients who did not perform the exercise in the hospital but at a local physiotherapy practice, the exact data on adherence were missing. The training log for the subsequent home-based unsupervised exercise was filled out by 14 of 29 patients. In group A, adherence to the home-based exercise was 77.8% (range 41.7–125.0%); in group B, adherence was 72.2% (range 5.6–138.9%), *P* = 0.947.

In total, 3 out of 30 patients (10.0%), all in group A compared to none in group B, developed signs of bleomycin-induced pulmonary toxicity (*P* = 0.224). Of these patients, two were previous smokers and one patient had never smoked. One patient developed clinical and radiographic signs of bleomycin-induced pulmonary toxicity during BEP chemotherapy, resulting in discontinuing bleomycin administration (after receiving 210 United States Pharmacopeia (USP)). One patient developed clinical signs of bleomycin-induced pulmonary toxicity during chemotherapy and radiographic signs after completion of chemotherapy (after receiving 270 USP, no dose reduction). One patient developed clinical and radiographic signs of bleomycin-induced pulmonary toxicity after completion of chemotherapy (cumulative bleomycin dose: 270 USP). Regarding occurrence of vascular events, none of the patients in group A developed a vascular event. Of the patients in group B, 2 out of 15 patients developed a vascular event (13.3%, *P* = 0.343). Of these patients, one developed pulmonary embolisms and one patient developed thrombosis of the basilar and ulnar veins, both occurred after completion of the second course of BEP chemotherapy.

No exercise-related (serious) adverse events occurred. One patient in group B developed pulmonary embolisms during chemotherapy. One patient in group A was hospitalized due to a Mycoplasma Pneumonia infection after completion of chemotherapy. One patient in group A had melena during chemotherapy, for which no cause was found with gastroduodenoscopy.

One patient in group B withdrew his consent during chemotherapy, because he thought that participating in the exercise intervention after completion of chemotherapy would be too time consuming.

**Fig. 3:**
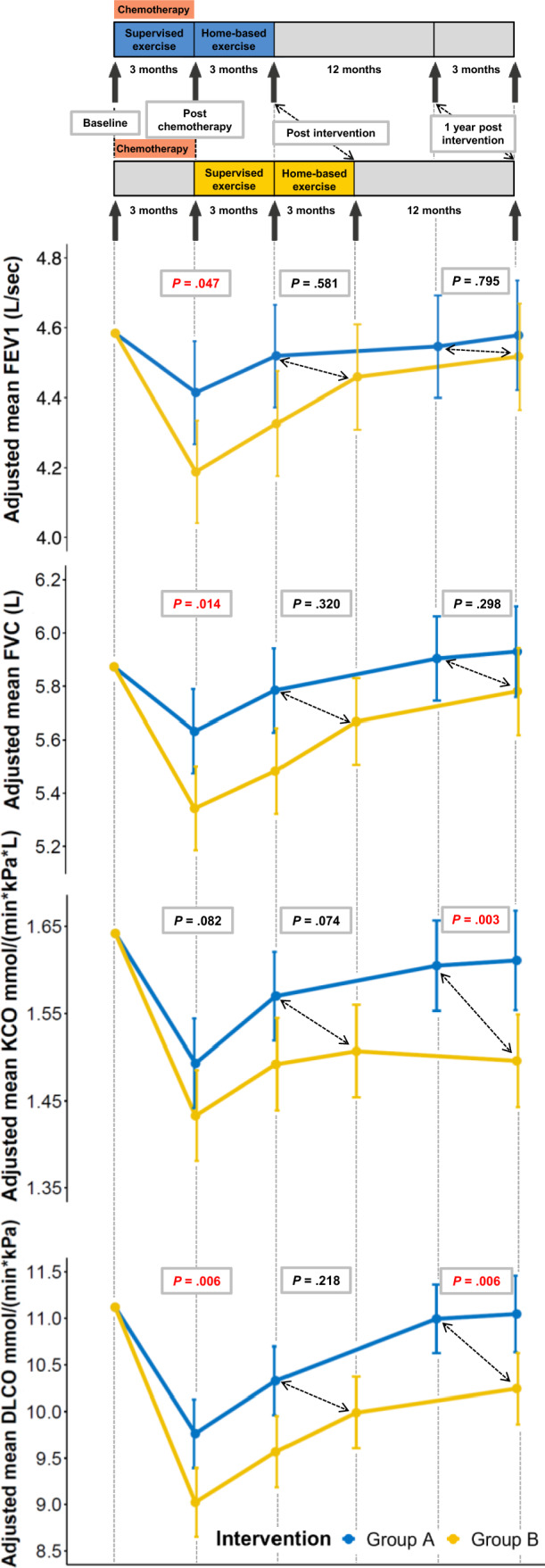
Effects of the tailored physical exercise intervention during or after BEP-chemotherapy on pulmonary function. *Group A* initiated the intervention during chemotherapy, *Group B* initiated the intervention after chemotherapy, *BEP* bleomycin, etoposide and cisplatin, *DLCO* carbon monoxide diffusing capacity, *KCO* carbon monoxide transfer coefficient, *FVC* forced vital capacity, *FEV1* forced expiratory volume-one second

Results of pulmonary function parameters are shown in Tables 2 and 3 and Fig. 3. Directly post-chemotherapy, exercise has led to significantly less decline in FVC%predicted and DLCO%predicted (mean between-group A and B difference 4.4, 95% CI 0.5–8.4, ES: 0.38, *P* = 0.029 and 5.7, 95% CI 0.9–10.4, ES: 0.54, *P* = 0.020, respectively). Comparable, but non-significant effects were found for FEV1%predicted and KCO%predicted (4.2, 95% CI – 0.3 to 8.7, ES: 0.37, *P* = 0.067, and 3.7 95% CI – 0.7 to 8.2, ES: 0.24, *P* = 0.099, respectively). The between-group difference of FEV1 (raw data) measured post-chemotherapy was statistically significant in favour of group A (0.22, 95% CI 0.0–0.4, p = 0.047, Fig. 3). After both groups had completed 24 weeks of exercise, no statistically significant differences were observed in the pulmonary function parameters between group A and B. At 1-year post-intervention, significantly smaller declines in KCO%predicted and DLCO%predicted were found in group A compared to group B (6.7, 95% CI 2.2–11.2, ES: 0.43, *P* = 0.004 and 5.4, 95% CI 0.7–10.2, ES: 0.52, *P* = 0.026, respectively).

**Table 2:** Pulmonary function parameters post-chemotherapy, post-intervention and 1 year post-intervention

	Baseline		Post-chemotherapy		Post-intervention		1 year post-intervention	
	Mean (SD)	*n*	Mean (SD)	*n*	Mean (SD)	*n*	Mean (SD)	*n*
FVCpredicted								
Group A	96.7 (12.5)	15	93.1 (10.4)	15	95.8 (10.0)	15	98.0 (11.1)	15
Group B	103.3 (11.0)	15	94.4 (14.1)	15	99.9 (10.6)	14	102.3 (12.5)	14
FEV1predicted								
Group A	91.9 (12.4)	15	88.9 (11.4)	15	91.2 (12.0)	15	92.5 (13.2)	15
Group B	99.1 (10.2)	15	90.5 (11.9)	15	96.9 (7.5)	14	98.4 (9.5)	14
FEV1/FVC								
Group A	77.8 (6.3)	15	78.3 (7.0)	15	77.9 (6.7)	15	76.8 (6.9)	15
Group B	78.5 (4.8)	15	78.9 (6.5)	15	79.6 (6.4)	14	78.8 (6.0)	14
DLCO%predicted								
Group A	90.7 (8.7)	15	79.5 (8.5)	15	84.6 (8.9)	15	92.6 (8.0)	15
Group B	88.3 (11.9)	15	72.1 (10.2)	15	81.4 (9.5)	14	86.3 (13.2)	14
KCO%predicted								
Group A	104.5 (18.6)	15	94.1 (9.0)	15	98.9 (13.4)	15	101.4 (12.7)	15
Group B	96.7 (12.0)	15	85.8 (9.9)	15	91.1 (10.8)	14	90.7 (11.0)	14

*group A* exercise intervention initiated during chemotherapy, *group B* exercise intervention initiated after chemotherapy, *SD* standard deviation, *FVC* forced vital capacity, *FEV1* forced expiratory volume-one second, *DLCO* carbon monoxide diffusing capacity, *KCO* carbon monoxide transfer coefficient

**Table 3:** Linear-mixed effects model results of pulmonary function parameters post-chemotherapy, post-intervention and 1 year post-intervention

	Within-group difference Post-chemotherapy	Within-group difference Post-intervention	Within-group difference 1 year post-intervention	Between-group difference Post-chemotherapy		Between-group difference Post-intervention		Between-group difference 1 year post-intervention	
	LSM difference (95% CI)	LSM difference (95% CI)	LSM difference (95% CI)	LSM difference (95% CI)	Effect size (– 1 to 1)	LSM difference (95% CI)	Effect size (– 1 to 1)	LSM difference (95% CI)	Effect size (– 1 to 1)
FVC%predicted									
Group A	– 3.6 (– 6.8 to – 0.4)	– 0.9 (– 5.2 to 3.5)	1.3 (– 3.8 to 6.4)	4.4 (0.5–8.4)	0.38^#^	1.7 (– 2.3 to 5.7)	0.14	1.6 (– 2.5 to 5.6)	0.13
Group B	– 8.9 (– 12.1 to – 5.7)	– 3.4 (– 7.8 to 1.0)	– 1.1 (– 6.3 to 4.1)						
FEV1%predicted									
Group A	– 3.0 (– 6.6 to 0.6)	– 0.7 (– 5.5 to 4.2)	0.6 (– 5.0 to 6.2)	4.2 (– 0.3 to 8.7)	0.37	0.7 (– 3.9 to 5.3)	0.06	0.2 (– 4.4 to 4.8)	0.02
Group B	– 8.5 (– 12.1 to – 4.9)	– 3.1 (– 8.0 to 1.8)	– 1.4 (– 7.1 to 4.3)						
FEV1/FVC									
Group A	0.5 (– 1.2 to 2.2)	0.1 (– 2.2 to 2.4)	– 1.0 (– 3.7 to 1.7)	0.03 (– 2.9 to 3.0)	0.00	– 0.3 (– 3.2 to 2.7)	– 0.05	– 0.6 (– 3.6 to 2.3)	– 0.1
Group B	0.4 (– 1.3 to 2.1)	0.3 (– 2.1 to 2.6)	– 0.5 (– 3.2 to 2.3)						
DLCO%predicted									
Group A	– 11.2 (– 15.1 to – 7.4)	– 6.1 (– 11.2 to – 1.1)	1.9 (– 3.8 to 7.6)	5.7 (0.9–10.4)	0.54^#^	2.2 (– 2.6 to 7.0)	0.21	5.4 (0.7–10.2)	0.52^#^
Group B	– 16.1 (– 20.0 to – 12.3)	– 7.3 (– 12.4 to – 2.1)	– 2.3 (– 8.1 to 3.6)						
KCO%predicted									
Group A	– 10.4 (– 14.6 to – 6.2)	– 5.7 (– 11.3 to – 0.09)	– 3.1 (– 9.6 to 3.4)	3.7 (– 0.7 to 8.2)	0.24	3.8 (– 0.7 to 8.4)	0.25	6.7 (2.2–11.2)	0.43^#^
Group B	– 10.9 (– 15.1 to – 6.7)	– 6.2 (– 11.9 to – 0.4)	– 6.3 (– 13.0 to 0.2)						

*group A* exercise intervention initiated during chemotherapy, *group B* exercise intervention initiated after chemotherapy, *FVC* forced vital capacity, *FEV1* forced expiratory volume-one second, *DLCO* carbon monoxide diffusing capacity, *KCO* carbon monoxide transfer coefficient, *LSM* least squares mean, *SE* standard error, *95%CI* 95% confidence interval

^#^Statistically significant (*p*-value < 0.05), Group A: physical exercise initiated during BEP chemotherapy, Group B: physical exercise initiated after BEP chemotherapy

*P*-value for mixed model between-group measures comparing changes in group A and B from baseline to post-chemotherapy, post-intervention, 1 year post-intervention, adjusted for baseline value (adjusted baseline values: FVC%predicted: 100.0, FEV1%predicted: 95.4, FEVFVC: 78.2, DLCO%predicted: 89.9, KCO%predicted: 101.0)

**Fig. 4:**
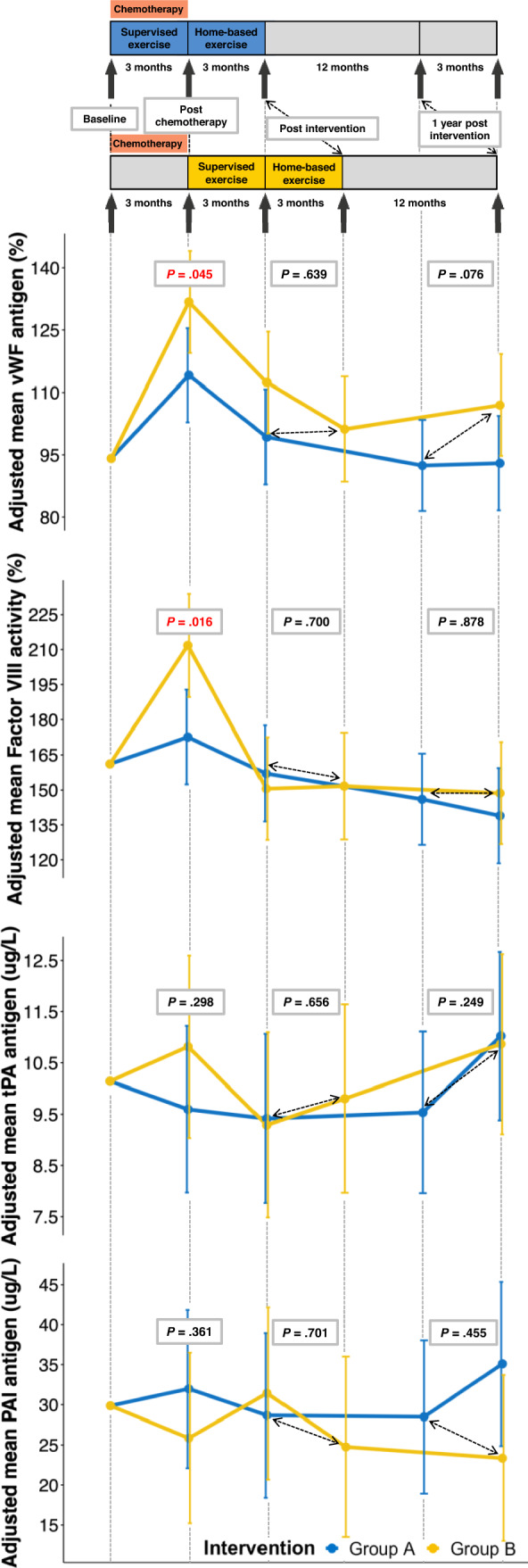
Effects of the tailored physical exercise intervention during or after BEP-chemotherapy on markers of vascular endothelial dysfunction. *Group A* initiated the intervention during chemotherapy, *Group B* initiated the intervention after chemotherapy, *BEP* bleomycin, etoposide and cisplatin, *vWF* von Willebrand factor, *PAI-1* plasminogen activator inhibitor type 1 antigen, *t-Pa* tissue-type plasminogen activator antigen

Directly post-chemotherapy, vWF and factor VIII increased significantly less in group A compared to group B (– 17.1%, 95% CI – 33.8% to – 0.4%, ES: -0.51, *P* = 0.045*.* and – 40.1%, 95% CI – 72.4% to – 7.8%, ES: – 0.77, *P* = 0.016, respectively) (Fig. 4, Supplemental Tables 2 and 3). Levels of fibrinogen and t-PA decreased non-significantly in group A, while patients in group B showed an non-significant increase in these markers directly post-chemotherapy (between-group difference: – 0.4, 95% CI – 0.8 to 0.1; ES: – 0.25, *P* = 0.138 and – 1.2, 95% CI – 3.6 to 1.1, ES – 0.36, *P* = 0.298, respectively). In group A, PAI-1 increased, while a decrease was observed in group B, with a non-significant between-group difference of 5.3, 95% CI – 6.2 to 16.8: ES 0.28, *P* = 0.361. Post-intervention and 1-year post-intervention, no between-group differences were found in all markers.

## Discussion

In this exploratory subgroup analysis of the ACT-trial, we found beneficial effects of a physical exercise intervention initiated during compared to after BEP-chemotherapy on pulmonary function parameters and markers of endothelial dysfunction in patients with metastatic testicular cancer. We found statistically significant differences between groups for FVC, FEV1, DLCO, KCO, vWF and factor VIII, measured directly post-chemotherapy in favour of the group that exercised during chemotherapy (group A). Also, 1-year post-intervention, significant differences were found in pulmonary function (KCO, DLCO) in favour of group A.

Physical exercise may reduce the BEP-induced generation of reactive oxygen species and thereby prevent the development of vascular endothelial dysfunction and the consequent inflammatory response (Simioni et al. 2018). In pulmonary tissue, this could manifest in better preservation of the permeability of the alveolar-capillary membrane (Plantier et al. 2018). This might explain the limited decline of DLCO in patients who attended the physical exercise intervention during chemotherapy in our study. Another explanation for the difference in DLCO between the groups could be that the respiratory muscle weakness due to chemotherapy, use of corticosteroids and immobility during hospitalization in group B was more profound, leading to submaximal inspiratory volumes and functional alveolar volumes (Graham et al. 2017). Since DLCO is the product of KCO and alveolar volume, a fall in alveolar volume may subsequently lead to a decrease in DLCO (Graham et al. 2017). However, a reduction in DLCO due to inspiratory muscle weakness is typically accompanied by a compensatory increase in KCO (Hughes and Pride 2001; Hart et al. 2002). We did not observe an increase in KCO, suggesting that the underlying cause of the difference in DLCO between both groups is more likely a difference in permeability of the alveolar-capillary membrane.

We also found that FEV1 and FVC were better preserved during chemotherapy in group A compared to group B. The physical exercise intervention during BEP-chemotherapy may maintain physical fitness and respiratory muscle strength and thereby possibly preserved values of FEV1 and FVC (Kenn et al. 2013; Vainshelboim et al. 2014). Regarding the effect of exercise on pulmonary function, as measured by FVC and FEV1, the results should be interpreted cautiously because of our small patient group and because of the study of Lauritsen et al. (2016), who did not detect any difference in FVC and FEV1 pre- to post BEP-chemotherapy among 550 patients with testicular cancer. Additionally, the absolute difference in pulmonary volumes in our study is small (post-chemotherapy FVC: 4.4%, FEV1 4.2%) and clinical relevance is questionable. Regarding DLCO, the study of Lauritsen et al. did find a statistically significant decline post BEP-chemotherapy compared to pre BEP-chemotherapy (11.1%, *P* < 0.001). Five years after completion of chemotherapy, the DLCO had returned to baseline values in these patients: diffusion capacity abnormality was detected in 15.6% (95% CI 11.3–19.9%) at 5 years follow-up compared with 20.7% pretreatment (95% CI 16.6–24.8%). Also, Lauritsen et al. found that after a median follow-up of 16.1 years, the 15-year cumulative risk of pulmonary diseases in patients treated with BEP-chemotherapy was comparable to that of patients with stage 1 testicular cancer who were observed on a surveillance program. Unfortunately, no data is shown about the level of physical activity during and after treatment with BEP-chemotherapy. In our study, patients in group A (exercise during BEP-chemotherapy) declined by 11.2% and patients in group B (exercise after BEP-chemotherapy) declined by 16.1%, with a statistically significant within-group difference of 5.7 (95% confidence interval 0.9–10.4, with a moderate effect size of 0.54). However, the study of Shamash et al. (2017) only showed a weak correlation between the decline in DLCO and pulmonary toxicity on a chest CT-scans. Therefore, DLCO may not be used as marker for pulmonary toxicity and it is questionable whether a decrease in DLCO should be used in the clinic to discontinue bleomycin). However, DLCO may be considered as marker for vascular endothelial dysfunction. A recent study investigating vascular endothelial function and pulmonary function in patients with type 2 diabetes, found a strong correlation between DLCO and vascular endothelial function parameters (nitric oxide, *r* = 0.797, endothelin-1, *r* = – 0.787 and flow mediated dilatation, *r* = 0.700) (3). In multiple regression analysis, DLCO remained significantly correlated with flow mediated dilatation, which is a well-known marker for endothelial function (Tai et al. 2021). The reduced decline in DLCO in patients who exercised during BEP-chemotherapy, combined with less increase in factor VIII and vWF in these patients, could be considered as a hypothesis-generating result, since all three markers are suggestive for vascular endothelial damage.

Results of the present study suggest that attending physical exercise during BEP-chemotherapy might protect against vascular endothelial dysfunction. Currently, limited data are available on effects of exercise on vascular endothelial dysfunction. A retrospective study in elderly male volunteers showed a significant inverse dose–response relationship between physical exercise and fibrinogen levels, factor VIII, vWF, t-Pa and PAI-1 (Wannamethee et al. 2002). A recent meta-analysis including four randomized controlled trials, indicated that exercise training improves vascular endothelial function in breast and prostate cancer survivors, measured with flow-mediated dilatation of the brachial artery or the reactive hyperemia index (Beaudry et al. 2018). Exercise increased the flow-mediated dilatation with 1.3%, as compared to non-exercise. Importantly, a 1% increase in flow-mediated dilatation is associated with a reduction of 8–13% of future cardiovascular events (Beaudry et al. 2018). Lubberts et al. found that vWF and factor VIII significantly increased in patients with metastatic testicular cancer during (B)EP-chemotherapy without participation in an exercise intervention, which lasted up to 1-year post-chemotherapy (2016). The increase in factor VIII and vWF during chemotherapy was comparable to the outcome of our study in group B. However, this increase was less profound in patients who started the supervised exercise intervention during BEP-chemotherapy.

In total, 3 out of 30 patients (10.0%, non-significant), all in group A, developed signs of bleomycin-induced pulmonary toxicity. Two out of 30 patients (13.3%, non-significant), all in group B, developed a vascular event. However, the small numbers make it difficult to draw conclusions about the clinical significance and importance of this finding. Future studies including larger patient cohorts attending physical exercise during and after BEP-chemotherapy, should monitor the occurrence of bleomycin-induced pulmonary toxicity and occurrence of vascular events.

No adverse events related to the exercise intervention occurred in our study. In a study in which patients with testicular cancer were randomized to supervised high-intensity interval training (HIIT, 85–95% of HR_peak_) during (B)EP-chemotherapy or a usual care group, three of the first nine patients randomized to HIIT developed severe thromboembolic events during the intervention (Thorsen et al. 2020). Possibly, HIIT increased vascular shear stress and causes relative dehydration, inducing an increase in blood viscosity (Wolberg et al. 2012). Since these are risk factors for developing thromboembolic events, HIIT might have played a role in developing thromboembolic events. In contrast, our study’s supervised physical exercise intervention comprised a tailored continuous endurance training with moderate-to-vigorous intensity and strength training, gradually increasing in intensity over the weeks. Results of another study that investigated HITT in patients with testicular cancer after completing their treatment suggest that HITT is safe and feasible after treatment (Adams et al. 2017).

Comparison of the effects of exercise on treatment-related morbidity to a control group would have been very interesting. However, when the study protocol of the ACT-trial was written (2012), it was already known that physical exercise during and after treatment with chemotherapy has evident beneficial effects on various chemotherapy-related adverse effects, and that physical training was beneficial on several outcomes such as physical fitness, muscle strength, fatigue, and quality of life. In addition, exercise programs appeared to be safe. However, knowledge of the timing of the physical exercise training was lacking and therefore, the topic of this study was randomizing exercise during versus after chemotherapy.

To our knowledge, this is the first study showing beneficial effects of physical exercise during BEP-chemotherapy on pulmonary function parameters and markers of vascular endothelial activation in patients with testicular cancer. The study had several strengths and limitations. Strengths are the randomized design and the high adherence rate to the physical exercise intervention. Limitations are the small patient groups and the lack of importance of DLCO in predicting bleomycin-induced pulmonary toxicity (Watson et al. 2018; Sleijfer 2001). However, DLCO may be used as marker of vascular endothelial dysfunction (Tai et al. 2021). Despite the limitations described above, the suggestion of a protective effect of a physical exercise intervention during BEP-chemotherapy on vascular endothelial function, warrants further research in larger patient groups with longer follow-up*.* For example, future studies investigating the effects of exercise during and after chemotherapy should investigate the occurrence of e.g. cardiovascular events and other cardiovascular related late effects in the longer term.

## Conclusion

In this explorative analysis, patients who started the exercise intervention during BEP-chemotherapy better preserved their pulmonary function (DLCO, FVC), measured directly post-chemotherapy and 1-year post-intervention (DLCO, KCO). This coincided with less increase in factor VIII and vWF, measured directly post-chemotherapy. These data support a beneficial role of a physical exercise intervention during BEP-chemotherapy on pulmonary and vascular toxicity in patients with testicular cancer and warrant further investigation. Three patients in group A developed BIP, compared to none in group B. Two patients in group B developed a vascular event, compared to none in group A. The small numbers of patients with BIP and vascular events warrant caution when interpreting these data.

## Supplementary Information

Below is the link to the electronic supplementary material.

Supplementary file1 (DOCX 30 KB)

## Data Availability

The datasets generated during and/or analyzed during the current study are available from the corresponding author on reasonable request.
