# Supplementary material for: Physical exercise in patients with testicular cancer treated with bleomycin, etoposide and cisplatin chemotherapy: pulmonary and vascular endothelial function—an exploratory analysis
Source: J Cancer Res Clin Oncol. 2023 Oct 27;149(19):17467–78. doi: 10.1007/s00432-023-05469-5 (PMC10657310; doi:10.1007/s00432-023-05469-5)
Supplement: Supplementary file 1 — Supplementary file1 (DOCX 30 KB) [file 432_2023_5469_MOESM1_ESM.docx]

**Supplementary material**

**Supplemental information**

*Exercise intervention*

The supervised exercise intervention consisted of three components: three times per week ~ 30 min of aerobic exercise training (AET) on a bicycle ergometer or treadmill, in combination with twice per week 20 to 30 min of resistance exercise training (RET), and once per week a game session, with sports like indoor hockey, badminton or soccer as addition to the aerobic exercise training. A trained physiotherapist personalized the intervention to the patients’ capabilities. The intensity of aerobic training was prescribed based on training heart rate (THR), calculated with the Karvonen formula (THR = (HR_max_ - HR_rest_) * intensity (%) + HR_rest_) ^39^, using the HR_max_ and HR_rest_ from the cardiopulmonary exercise testing (CPET) assessed at baseline. The AET intensity increased over the ensuing weeks (week 1-4: 50%, week 5-6: 60%, week 7-10: 70%, and week 11-12: 75%). RET consisted of training large skeletal muscles with the following exercises: leg press, seated row, chest press, abdominal crunch, leg curl, leg extension. RET intensity was fixed on 50% of the 1-repetition maximum (1-RM) during the first week. The intensity increased by 5%-10% during the following weeks, and 1-RM was again determined in week 7. Physiotherapists monitored attendance, adverse events, and intensity of the training. After completion of the supervised intervention, a 12-week, home-based, unsupervised aerobic exercise program was provided. During this period, patients were asked to continue AET home-based by walking, running, or cycling. The level of intensity was 60%-75%, according to the Karvonen formulae. Patients were requested to record their adherence to the exercise in a training log in the same way as during the supervised intervention. The supervised exercise intervention consists of 18.95 MET-h/week (based on an average person weighing 70 kg, using the 2011 Compendium of Physical Activities): bicycle stationary (moderate to vigorous effort): 6.8 METs * 1.5 hours: 10.2 MET-h, resistance training (weight lifting, free weight, nautilus or universal): 6 METs * 1 hours: 6 MET-h and badminton (social singles or doubles), general: 5.5 METs * 0.5 hours: 2.75 MET-h.

Supplementary Table 1. An exemplar exercise schedule

| Day | Activity | Duration |
| --- | --- | --- |
| Monday |  |  |
|  | aerobic exercise training on a bicycle ergometer or treadmill | 30 minutes |
|  | resistance exercise training (large skeletal muscles with the following exercises: leg press, seated row, chest press, abdominal crunch, leg curl, leg extension) | 20 – 30 minutes |
| Wednesday |  |  |
|  | aerobic exercise training on a bicycle ergometer or treadmill | 30 minutes |
|  | game session, with sports like indoor hockey, badminton or soccer | 30 minutes |
| Friday |  |  |
|  | aerobic exercise training on a bicycle ergometer or treadmill | 30 minutes |
|  | resistance exercise training  (large skeletal muscles with the following exercises: leg press, seated row, chest press, abdominal crunch, leg curl, leg extension) | 20 – 30 minutes |

**Supplementary Table 2: Vascular endothelial damage parameters post-chemotherapy, post-intervention and 1 year post-intervention**

|  | Baseline | | Post-chemotherapy | | Post-intervention | | 1 year post-intervention | |
| --- | --- | --- | --- | --- | --- | --- | --- | --- |
|  | Mean (SD) | n | Mean (SD) | n | Mean (SD) | n | Mean (SD) | n |
| Factor VIII | |  |  |  |  |  |  |  |
| Group A  Group B | 159.0 (53.5) 161.7 (49.2) | 15  13 | 172.0 (45.3)  206.7 (78.6) | 14  13 | 156.8 (32.9)  150.1 (49.0) | 13  12 | 144.7 (42.3)  151.1 (49.7) | 15  14 |
| VWF | |  |  |  |  |  |  |  |
| Group A  Group B | 91.6 (40.1) 97.5 (23.0) | 15  13 | 112.5 (31.8)  125.3 (27.0) | 14  13 | 96.6 (21.7)  106.6 (29.6) | 13  12 | 91.0 (27.3)  109.3 (28.5) | 14  13 |
| Fibrinogen | |  |  |  |  |  |  |  |
| Group A  Group B | 3.3 (1.7)  2.8 (0.9) | 15  13 | 3.0 (0.5)  3.3 (0.8) | 14  13 | 2.5 (0.3)  2.8 (1.0) | 13  12 | 2.6 (0.5)  2.6 (0.6) | 15  14 |
| PAI-1 | |  |  |  |  |  |  |  |
| Group A  Group B | 24.1 (9.9)  37.2 (24.5) | 14 13 | 28.9 (15.6)  26.1 (17.0) | 14 13 | 24.4 (16.8)  28.7 (20.6) | 13  12 | 24.4 (13.0)  26.7 (18.1) | 15  14 |
| t-PA | |  |  |  |  |  |  |  |
| Group A  Group B | 9.7 (2.6)  10.7 (4.2) | 15  13 | 9.4 (2.6)  10.3 (4.8) | 14  13 | 9.0 (2.4)  10.0 (3.7) | 13  12 | 9.2 (2.7)  10.8 (5.0) | 15  14 |

Abbreviations: group A: exercise intervention initiated during chemotherapy, group B: exercise intervention initiated after chemotherapy, SD: standard deviation, vWF; von Willebrand factor, PAI-1; plasminogen activator inhibitor type 1 antigen, t-Pa; tissue-type plasminogen activator antigen.

**Supplementary Table 3:** **Linear-mixed effects model results of vascular endothelial damage parameters post-chemotherapy, post-intervention and 1 year post-intervention**

|  | Within-group difference  Post-chemotherapy | Within-group difference  Post-intervention | Within-group difference  1 year post-intervention | Between-group difference  Post-chemotherapy | | Between-group difference  Post-intervention | | Between-group difference  1 year post-intervention | |
| --- | --- | --- | --- | --- | --- | --- | --- | --- | --- |
|  | LSM difference (95% CI) | LSM difference (95% CI) | LSM difference (95% CI) | LSM difference (95% CI) | Effect size  (-1 to 1) | LSM difference (95% CI) | Effect size  (-1 to 1) | LSM difference (95% CI) | Effect size  (-1 to 1) |
| Factor VIII |  |  |  |  |  |  |  |  |  |
| Group A  Group B | 12.2 (-9.1 to 33.5)  49.8 (26.7 to 72.9) | -3.4 (-30.9 to 24.0)  -13.1 (-42.0 to 15.7) | -14.3 (-44.0 to 15.5)  -8.9 (-40.1 to 22.4) | -40.1  (-72.4 to  -7.8) | -0.77# | 6.4  (-26.5.0 to 39.2) | 0.12 | -2.4  (-33.8 to 29.0) | -0.05 |
| VWF |  |  |  |  |  |  |  |  |  |
| Group A  Group B | 21.0 (10a.2 to 31.7)  33.4 (21.7 to 45.1) | 6.3 (-7.9 to 20.5)  10.0 (-4.9 to 25.0) | -0.7 (-16.5 to 15.1)  14.7 (-1.9 to 31.3) | -17.1  (-33.8 to  -0.4) | -0.51# | -4.0  (-20.8 to 12.9) | -0.12 | -14.8  (-31.2 to 1.6) | -0.44 |
| Fibrinogen |  |  |  |  |  |  |  |  |  |
| Group A  Group B | -0.3 (-0.9 to 0.3)  0.5 (-0.1 to 1.1) | -0.8 (-1.4 to -0.1)  -0.01 (-0.7 to 0.7) | -0.6 (-1.2 to 0.01)  -0.2 (-0.9 to 0.5) | -0.4  (-0.8 to 0.1) | -0.25 | -0.5  (-0.9 to 0.4) | -0.32 | -0.04  (-0.5 to 0.4) | -0.03 |
| PAI-1 |  |  |  |  |  |  |  |  |  |
| Group A  Group B | 4.3 (-4.0 to 12.5)  -7.0 (-15.7 to 1.6) | -0.3 (-10.6 to 10.0)  -8.5 (-19.1 to 2.2) | 0.4 (-10.5 to 11.3)  -9.5 (-20.8 to 1.8) | 5.3  (-6.2 to 16.8) | 0.28 | 2.4  (-9.8 to 14.5) | 0.13 | 4.3  (-7.1 to 15.6) | 0.23 |
| t-PA |  |  |  |  |  |  |  |  |  |
| Group A  Group B | -0.4 (-2.0 to 1.2)  0.4 (-1.4 to 2.1) | -0.6 (-2.7 to 1.5)  -0.4 (-2.6 to 1.8) | -0.4 (-2.7 to 1.5)  0.4 (-1.9 to 2.8) | -1.2  (-3.6 to 1.1) | -0.36 | -0.6  (-3.0 to 1.9) | -0.16 | -1.3  (-3.6 to 1.0) | -0.38 |

Abbreviations: group A: exercise intervention initiated during chemotherapy, group B: exercise intervention initiated after chemotherapy, vWF; von Willebrand factor, PAI-1; plasminogen activator inhibitor type 1 antigen, t-Pa; tissue-type plasminogen activator antigen, LSM: least squares mean, SE: standard error, 95%CI: 95% confidence interval, P-value for mixed model between-group measures comparing changes in group A and B from baseline to post-chemotherapy, post-intervention, 1 year post-intervention, adjusted for baseline values (adjusted baseline values: factor VIII: 161.4, vWF: 94.4, fibrinogen: 3.1, PAI-1: 29.5. t-Pa: 10.1). #: statistically significant (p-value < 0.05).
